# Twenty-year trends in cognitive performance and modifiable dementia risk factors: a Swiss population-based study

**DOI:** 10.1093/eurpub/ckag046

**Published:** 2026-03-31

**Authors:** Stephanie Schrempft, Claire Chevalier, Delia Antille, Roxane Dumont, Idris Guessous, Mayssam Nehme

**Affiliations:** Division of Primary Care Medicine, Geneva University Hospitals, Geneva, Switzerland; Division of Primary Care Medicine, Geneva University Hospitals, Geneva, Switzerland; Division of Geriatrics, Rehabilitation and Geriatrics Department, Geneva University Hospitals, Geneva, Switzerland; Division of Primary Care Medicine, Geneva University Hospitals, Geneva, Switzerland; Division of Primary Care Medicine, Geneva University Hospitals, Geneva, Switzerland; Department of Health and Community Medicine, Faculty of Medicine, University of Geneva, Geneva, Switzerland; Division of Primary Care Medicine, Geneva University Hospitals, Geneva, Switzerland; Department of Health and Community Medicine, Faculty of Medicine, University of Geneva, Geneva, Switzerland

## Abstract

Population trends in cognitive performance have been mixed, and few studies included data following the COVID-19 pandemic. We examined 20-year cognitive performance trends, and the contribution of modifiable dementia risk factors. We used repeated cross-sectional survey data from 2005 to 2025. Adults aged 50 to 75 years completed the clock drawing test (*N = *6902, 51% women). We calculated the updated LIfestyle for BRAin health (LIBRA2) index. Analyses used linear regression models. Cognitive performance declined over time [*β* (95% CI), −0.11 (−0.14 to −0.09), *P* <.001]. Higher LIBRA2 scores were associated with poorer cognitive performance [−0.08 (−0.11 to −0.05), <.001]. The decline in cognitive performance remained significant after adjustment for the LIBRA2 and its components. There was a small but significant decline in cognitive performance between 2005 and 2025. Well-known dementia risk factors did not fully explain the decline. Further research is needed to identify contributing factors, including the impact of digitalisation on society.

## Introduction

In the context of population ageing, whether today’s older adults are healthier than their predecessors is a key concern for individuals, families, researchers, clinicians, and policy makers. According to the ‘failure of success’ hypothesis, higher survival rates of individuals with health problems results in worse overall health of the older population [1]. In contrast, ‘the success-of-success’ hypothesis argues that factors contributing to decreased mortality also postpone disability onset, resulting in more people living longer and healthier lives [2].

To date, there is evidence that dementia incidence is declining in some high-income Western countries including the US, UK, and Sweden [3]. This decline is in part attributable to reduced rates of smoking and better education; while obesity and diabetes prevalence has increased [4]. It is estimated that 40% or more of dementias are preventable by improving modifiable risk factors such as low education, smoking, physical inactivity, obesity, diabetes, hypertension, hearing impairment, depression, and low social contact [5].

The ‘LIfestyle for BRAin health’ (LIBRA) was developed to identify individuals at increased risk of cognitive decline, and to motivate lifestyle changes for healthy brain ageing [6]. The LIBRA has previously been shown to predict cognitive functioning, cognitive impairment, structural brain changes, and incident dementia in several population-based cohort studies [6, 7]. The latest version of the index (LIBRA2) incorporates information from 15 modifiable risk and protective factors for dementia [6, 7], and is useful for monitoring lifestyle-based prevention potential in the general population.

There has been concern that the COVID-19 pandemic increased the burden of cognitive impairment among older adults due to direct effects of the virus [8], as well as adverse psychosocial environments that impacted health behaviours and mental health [9]. A recent US study found that the rate of dementia increased slightly at the onset of the COVID-19 pandemic, and mortality among those with dementia also rose sharply with the pandemic onset. Consequently, the downward trend in dementia prevalence for 2011–21 was even steeper than the pre-pandemic trend [10].

Secular trends are less clear when considering earlier stages on the spectrum of cognitive functioning. Studies on population trends prior to the COVID-19 pandemic have reported improvements or stability in cognitive functioning over time [11–13], while others have found a modest decline in cognitive functioning [14–16] or an increase in cognitive impairment [17]. Research on population trends during the pandemic found that cognitive performance declined from 2020 to 2022, and this decline was associated with changes in known dementia risk factors, including physical activity, alcohol consumption, and depression [9]. Little research has examined cognitive performance trends beyond the pandemic, and there is a need for continued tracking of cognitive health and associated risk factors over time.

In this representative cohort study of Swiss adults, we aimed to provide further insight into cognitive performance trends over the last 20 years, and the contribution of modifiable risk factors for dementia. We focused on the role of individual risk factors, as well as their combined role, using the LIBRA2 index.

## Methods

### Participants and study design

We used a repeated annual cross-sectional design, using independent samples of non-institutionalised individuals taking part in the Bus Santé population-based study in Geneva, Switzerland. Participants (aged 35–74 years until 2011, and 20–74 years afterwards) were selected each year from 1992, with a break during the pandemic (2020–22). Participants are selected using an annual list established by the local government and stratified random sampling by sex and 10-year age group. Those who are unreachable after three mailings and seven phone calls are replaced using the same selection procedure, but those who are reached and unwilling to participate are not replaced. Included participants are ineligible for future surveys. Of those contacted during the study period (2005–25), 49% participated. There was no difference between those contacted and those who participated in terms of age and sex (50% were women, and the mean age was 47 years for each group). We used data from 2005, when the clock test was introduced to individuals aged 50 years and above. To maximise power, we grouped survey years as follows: 2005–10, 2011–13, 2014–16, 2017–19, and 2023–25 (post-pandemic). We used 2-year time intervals, but combined information from 2005 to 2010 as there was a decrease in participant recruitment during 2005–08 due to another study taking place, which shared logistical resources but not the same target population. In sensitivity analyses using individual years, the overall trend in clock test performance was the same as that when using the 2-year time intervals.

All participants provided written informed consent, and the study was approved by the local institutional review board (IRB00003116). All methods and procedures were performed in accordance with the 1964 Declaration of Helsinki and its later amendments.

### Measures

Information on sociodemographic factors, health behaviours, and medical history was collected using standardised self-report questionnaires. Health examinations were conducted by trained research nurses. Body height and weight were measured using standard procedures, and body mass index (BMI) (kg/m^2^) was calculated. Blood pressure was measured thrice in the sitting position on the right arm after at least 10 minutes rest using an automated oscillometric sphygmomanometer. Venous blood samples were drawn after an overnight fast. Glucose, total plasma cholesterol, high-density lipoprotein (HDL) plasma cholesterol, and triglycerides were assayed using commercially available enzymatic kits (Bayer Technicon Diagnostics, CV 1.4%, 1.2%, and 1.5% for glucose, cholesterol, and triglycerides, respectively). LDL cholesterol was calculated using the Friedewald equation.

#### Cognitive performance

The clock drawing test is a cognitive screening instrument that taps into various cognitive skills including visuo-constructive abilities, verbal understanding, and memory [18]. The test is quick and easy to administer and score, is well accepted by participants, and correlates strongly with other measures of cognitive dysfunction, such as the Mini-Mental State Examination (MMSE) [18]. Participants were instructed to draw a clock, starting with a circle and inserting all the numbers, then setting the hands to ‘10 past 11’. These instructions could be repeated once more if needed. A score was calculated using Rouleau *et al.*’s [19] quantitative rating scale, which is based on the representation of the clockface (2 points), the layout of numbers (4 points), and the position of the hands (4 points). The total score ranges from 0 to 10, with higher scores indicating better cognition. A cutoff score of ≤7 has been used to identify cognitive impairment [20]. The clock tests were scored by the same psychologist throughout the study period, with good intra-rater reliability (see Supplemental Material).

#### Risk and protective factors

We calculated an aggregate measure of modifiable risk and protective factors for cognitive decline and dementia, based on the LIBRA2 index. The LIBRA2 comprises 15 modifiable risk factors for cognitive decline and dementia. Each risk and protective factor is assigned a weight based on risk estimates from previously published meta-analyses (eTable 1 in Supplemental Material). The weights are standardised and summed up to calculate the LIBRA2 score, with higher scores representing an unhealthier lifestyle.

Twelve of the 15 LIBRA2 risk and protective factors were available in our study, including high cognitive activity, low social contact, current smoking, high alcohol consumption (>21 units per week), high physical activity, healthy diet, obesity (BMI ≥30), diabetes, hypertension, high cholesterol, and coronary heart disease. Information on depression was available for a subsample of participants from 2012–19 and 2023–25 (*N = *1775), with a sub-analysis conducted for this risk factor. Information was not available for chronic kidney disease, hearing impairment, or sleep disturbances.

Cognitive leisure activities were conscious and intellectual activities and included writing, playing musical instruments, singing, dancing, sewing, and painting and repair. As reading, watching television, and playing cards were grouped together in the questionnaire, and TV viewing is a more passive cognitive activity that has been associated with cognitive decline in some studies [21], we created an aggregate score (hours per week) excluding these activities. Scores were divided into tertiles, with the highest tertile reflecting high cognitive activity. We used marital status (having never been married or lived as a couple) as a marker of low social contact [22]. Physical activity was measured using a Physical Activity Frequency Questionnaire (PAFQ), described in detail elsewhere [23]. For each type of activity [sedentary (<2 metabolic equivalent of tasks—METs), light (2 to <3 METs), moderate (3–6 METs), and vigorous (>6 METs)], the time spent per week was computed as average minutes per day × number of days performing the activity. High physical activity was defined as engaging in moderate activity for at least 150 minutes a week [24]. Dietary intake and alcohol consumption were assessed using a self-administered, semi-quantitative food frequency questionnaire (FFQ), which has been validated in the Geneva population and is described in detail elsewhere [25]. Mediterranean dietary scores were computed according to Trichopoulou *et al.* [26], excluding alcohol consumption, with higher scores representing a healthier diet. Scores were divided into tertiles, and the highest tertile reflected a healthy diet. Diabetes was fasting plasma glucose ≥7.0 mmol/L or self-reported diagnosis of diabetes. Hypertension was mean blood pressure ≥140/90 mmHg or self-reported diagnosis of hypertension. High cholesterol was total blood cholesterol ≥5 mmol/L and low density lipoprotein ≥ 3 mmol/L or self-reported diagnosis of hypercholesterolaemia. Coronary heart disease was a self-reported diagnosis of angina, atherosclerosis, or myocardial infarction. Probable depressive disorder was determined using the Patient Health Questionnaire-2 (PHQ-2), which is a validated measure of core symptoms of depression [27]. A total score of 3 or higher indicates probable depressive disorder.

Information on education level and last-known occupational position were additionally available and included as markers of cognitive engagement. Education level was coded as primary (compulsory education, typically completed aged 15–16 years in Switzerland), secondary (post-compulsory education before university or professional training, typically completed aged 18–19 years in Switzerland), and tertiary (university/professional level, usually completed after 18–19 years). Last-known occupational position was coded as executive or self-employed in a non-manual occupation, and employee or self-employed in a manual occupation.

#### Covariates

Covariates were age (in years) and biological sex (male, female). We also examined fatigue, self-rated health, and performance-based tests of physical function, which tend to covary with cognitive performance measures, and have been identified as early indicators of cognitive decline [28, 29]. Fatigue was measured as feeling generally weak, tired, or lacking in energy during the past 4 weeks (not at all, somewhat, a lot). Self-rated health was measured on a 5-point scale (1 = very bad, 5 = very good)—the last two categories (bad, very bad) were combined due to few cases reporting very bad health. The grip strength test was performed three times on the right hand, using a hydraulic hand dynamometer (Baseline Evaluation Instruments, New York). We took the mean of the three measurements. For the chair stand test, participants were asked to stand up and sit down on a firm chair without using their arms as quickly as they could for five rises. We used the time taken to complete the five chair stands (in seconds).

### Statistical analysis

We used linear regression models to estimate time effects (trends) for clock test scores, with time period modelled as a categorical variable every 2 years (with 2005–10 as the reference). To examine whether trends were driven by population improvements in risk factors, we ran this model adjusted for age and sex, then additionally adjusted for the LIBRA2 index. We also included the individual factors in a stepped approach, as follows: (i) educational attainment, last-known occupational position, and cognitive activity, (ii) social contact, (iii) smoking, alcohol consumption, physical activity, and diet quality, (iv) cardiovascular biomarkers, and (v) fatigue, general self-rated health, grip strength, and chair stand test time. We also ran minimally adjusted models (age and sex) for each modifiable risk factor. To test if trends vary by sex and age group, we estimated interactions between sex/age group and time. We repeated the analyses using logistic regression models, with cognitive impairment (yes/no) as the outcome. In sensitivity analyses, we applied sampling weights for each time period to maximise age and sex representativeness of the Geneva population. We also standardised the clock test scores by age and sex (of the entire study sample rather than to time-specific characteristics, since the distribution of age showed variability over the years). The results using either approach did not change, therefore we present results using the raw data. Lastly, we excluded cognitive activity from the LIBRA index (as better cognitive performance may lead to more cognitive engagement), but we saw no change in the results. For the analysis of overall trends in cognitive performance, we included all of those with data on the clock test and key covariates (age and sex; *N = *6902). For the analysis of the contribution of the LIBRA index to observed trends, we included those with data on all of the modifiable dementia risk factors [as the calculation of the index requires that data are available for all components; *N = *6232 (90%)].

For the sub-analysis with depression data, we ran the regression models with time modelled as a dichotomous variable [pre-pandemic (2012–19, *N = *716) versus post-pandemic (2023–25, *N = *1059)], and depression, age, and sex as covariates.

Data were analysed using Stata V.16.1 (Stata Corp, College Station, TX, USA).

## Results

**Figure 1.**
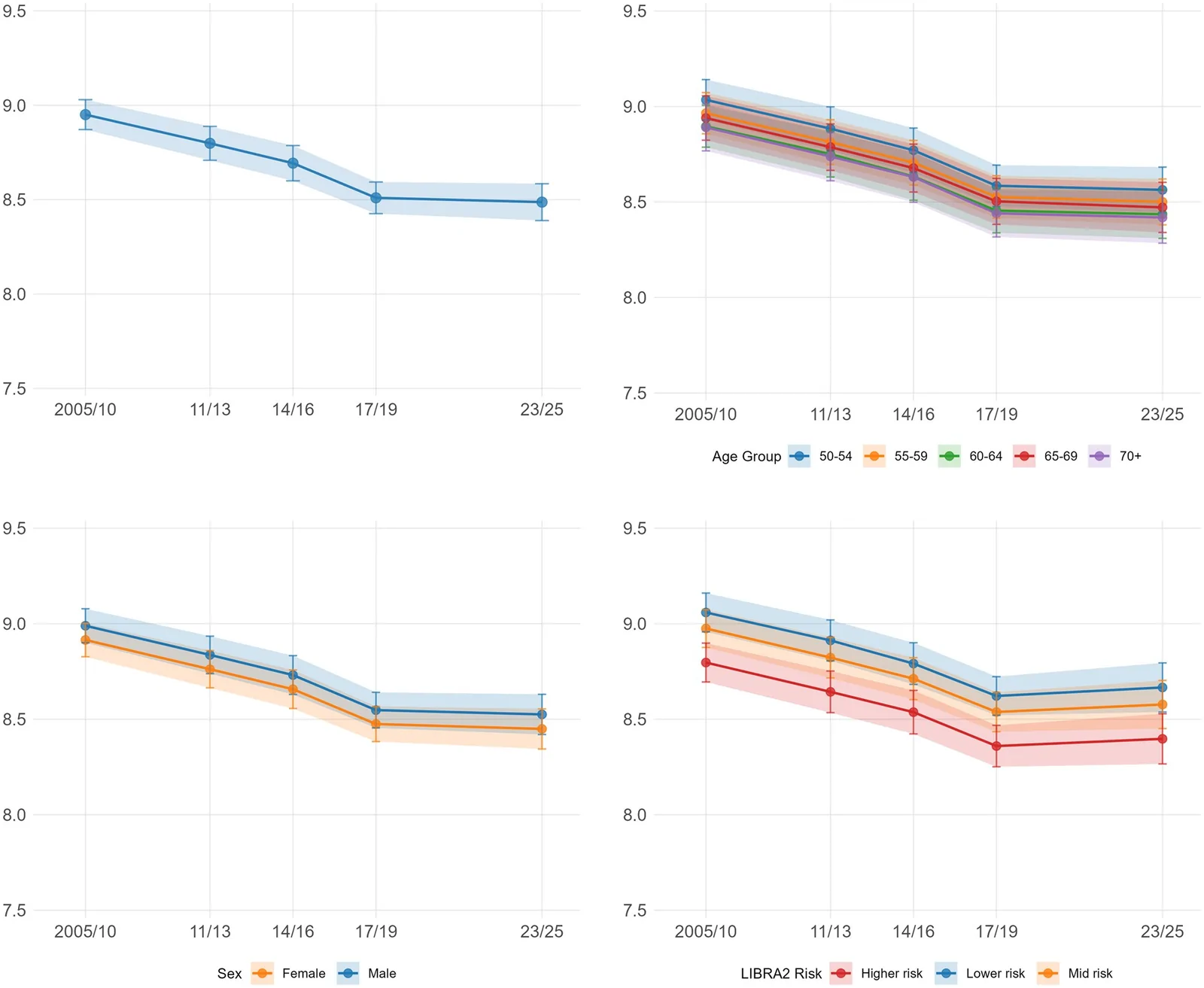
Mean clock test scores and 95% confidence intervals from 2005 to 2025 for the total sample and stratified by age group, sex, and LIBRA2 tertiles (*N = *6902).

Participant characteristics for the total sample (*N = *6902) and each time period are shown in Table 1 (eTable 2 in Supplemental Material for descriptives of all modifiable dementia risk factors examined in the study). Participants were on average 61 years old (SD 7.2), 51% were women, and 44% were educated to tertiary level. Mean (SD) clock test scores were 9.0 (1.5) during 2005–10, and 8.5 (1.8) during 2023–25, while the proportion of those with cognitive impairment was 14.2% (95% CI, 12.6–15.9) during 2005–10 and 27.4% (24.8–30.1) during 2023–25. As shown in Fig. 1, the decline in cognitive performance over time was observed across age and sex groups, and LIBRA2 scores (eFigs 1 and 2 in Supplemental Material for trends across individual risk and protective factors).

**Table 1.** Characteristics of Bus Sante participants with clock test scores during 2005–25^a^

	Total	2005/10	2011/13	2014/16	2017/19	2023/25
	*N = *6902	*N = *1701	*N = *1330	*N = *1226	*N = *1522	*N = *1123
Age in years	60.9 (7.2)	60.8 (6.8)	61.8 (7.7)	60.6 (7.1)	60.8 (7.2)	60.4 (7.1)
Age group						
50–54	24.3 (1674)	23.3 (397)	22.7 (302)	24.6 (302)	25.6 (390)	25.2 (283)
55–59	22.8 (1572)	22.0 (374)	19.6 (261)	25.0 (307)	22.7 (345)	25.4 (285)
60–64	19.8 (1370)	23.3 (397)	19.5 (260)	16.9 (207)	18.8 (286)	19.6 (220)
65–69	17.7 (1221)	17.8 (302)	20.5 (273)	18.9 (232)	16.3 (248)	14.8 (166)
70+	15.4 (1065)	13.6 (231)	17.6 (234)	14.5 (178)	16.6 (253)	15.0 (169)
Sex						
Male	48.7 (3360)	48.0 (817)	49.1 (653)	48.9 (599)	48.0 (731)	49.9 (560)
Female	51.3 (3542)	52.0 (884)	50.9 (677)	51.1 (627)	52.0 (791)	50.1 (563)
Education level						
Tertiary	44.0 (2966)	36.8 (613)	41.7 (545)	43.1 (523)	48.4 (724)	53.1 (561)
Secondary	41.9 (2822)	47.3 (789)	43.1 (564)	43.1 (523)	40.1 (601)	32.6 (345)
Primary	14.2 (955)	15.9 (266)	15.2 (199)	13.8 (167)	11.5 (172)	14.3 (151)
Occupational position						
Non-manual	81.0 (5352)	77.3 (1310)	80.6 (1067)	80.8 (987)	84.2 (1269)	83.5 (719)
Manual	19.0 (1256)	22.7 (384)	19.4 (257)	19.2 (235)	15.8 (238)	16.5 (142)
LIBRA2 score	1.3 (3.0)	1.6 (3.0)	1.4 (3.0)	1.1 (3.1)	1.0 (3.1)	1.2 (2.8)
Clock test score, range 0–10	8.7 (1.7)	9.0 (1.5)	8.8 (1.7)	8.7 (1.6)	8.5 (1.8)	8.5 (1.8)
Cognitive impairment						
No, clock test score ≥7	78.6 (5423)	85.8 (1459)	79.5 (1058)	78.7 (965)	74.0 (1126)	72.6 (815)
Yes, clock test score <7	21.4 (1479)	14.2 (242)	20.5 (272)	21.3 (261)	26.0 (396)	27.4 (308)

a Data are presented as mean (SD) for continuous measures, and % (*N*) for categorical measures.

**Figure 2.**
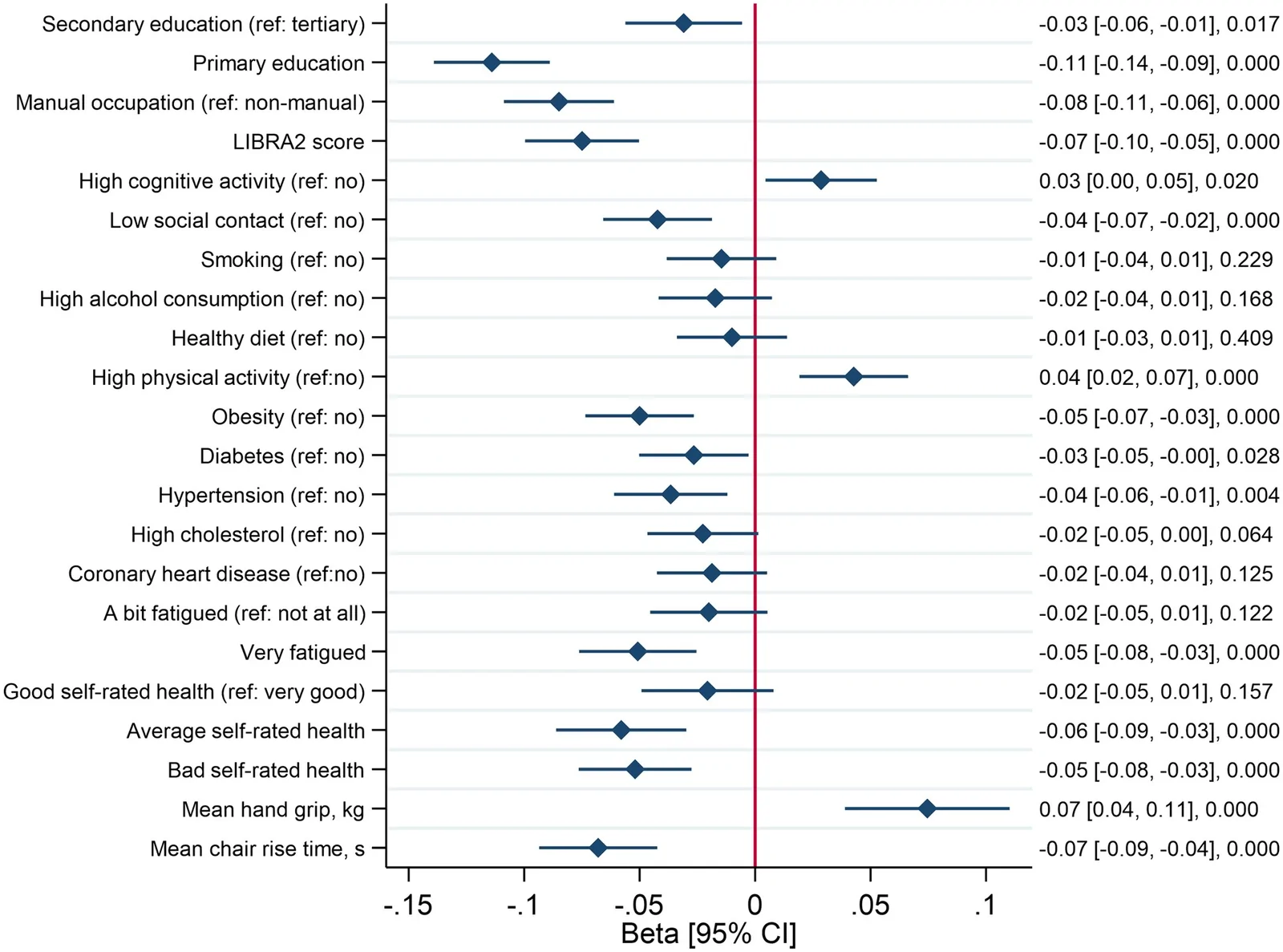
Associations between modifiable dementia risk factors and cognitive performance in age and sex-adjusted models.

Higher risk scores on the LIBRA2 index were significantly associated with poorer clock test performance in age- and sex-adjusted models [*β* (95% CI) = −0.08 (−0.10 to −0.05), *P* < .001]. Individual modifiable risk factors associated with clock test performance included cognitive activity (as well as education and occupational position), social contact, physical activity, obesity, diabetes, and hypertension (Fig. 2). Fatigue, poorer self-rated health, weaker grip strength, and slower chair rise time were also associated with poorer clock test performance (Fig. 2). The declining trend in clock test performance remained significant after adjustment for age, sex, the LIBRA2 index, and individual modifiable dementia risk factors and other health markers (Table 2 and eTable 3 in Supplemental Material). In the sub-analysis with depression, there was a decline in clock test scores from pre- to post-pandemic [*β* (95% CI) = −0.09 (−0.14 to −0.05), *P* < .001], and an increase in cognitive impairment [OR (95% CI) = 1.49 (1.18 to 1.87), *P* = .001], but there was no significant increase in depression from pre- to post-pandemic [OR (95% CI) = 1.48 (0.95 to 2.29), *P = .*081], nor an association between depression and clock test scores [*β* (95% CI) = −0.01 (−0.05 to 0.04), *P = .*774] or between depression and cognitive impairment [OR (95% CI) = 0.99 (0.63 to 1.58), *P = .*979].

**Table 2.** Adjusted clock test means and standard deviations over time for all models, with beta and 95% confidence intervals^a^

	Model 1		Model 2		Model 3		Model 4		Model 5		Model 6	
Time	*B* (95% CI)*P*	Mean (95% CI)	*B* (95% CI)*P*	Mean (95% CI)	*B* (95% CI)*P*	Mean (95% CI)	*B* (95% CI)*P*	Mean (95% CI)	*B* (95% CI)*P*	Mean (95% CI)	*B* (95% CI)*P*	Mean (95% CI)
2005/10 (REF)		8.95 (8.87 to 9.03)		8.96 (8.88 to 9.03)		8.96 (8.88 to 9.03)		8.96 (8.87 to 9.04)		8.95 (8.87 to 9.03)		9.03 (8.94 to v9.12)
2011/13	−0.15 (−0.27 to −0.03) .017	8.80 (8.71 to 8.89)	−0.16 (−0.28 to −0.04) .008	8.80 (8.71 to 8.89)	−0.16 (−0.28 to −0.04) .007	8.80 (8.71 to 8.89)	−0.15 (−0.27 to −0.03) .014	8.81 (8.72 to 8.90)	−0.16 (−0.28 to −0.03) .014	8.81 (8.72 to 8.90)	−0.25 (−0.38 to −0.12) .000	8.80 (8.71 to 8.89)
2014/16	−0.26 (−0.38 to −0.14) .000	8.69 (8.60 to 8.79)	−0.28 (−0.40 to −0.16) .000	8.70 (8.60 to 8.79)	−0.28 (−0.40 to −0.16) .000	8.70 (8.60 to 8.79)	−0.28 (−0.41 to −0.16) .000	8.69 (8.59 to 8.78)	−0.29 (−0.42 to −0.16) .000	8.69 (8.59 to 8.78)	−0.39 (−0.52 to −0.26) .000	8.68 (8.58 to 8.77)
2017/19	−0.44 (−0.56 to −0.33) .000	8.51 (8.43 to 8.59)	−0.47 (−0.58 to −0.35) .000	8.53 (8.44 to 8.61)	−0.47 (−0.58 to −0.35) .000	8.53 (8.44 to 8.61)	−0.44 (−0.56 to −0.33) .000	8.54 (8.45 to 8.63)	−0.46 (−0.58 to −0.34) .000	8.55 (8.46 to 8.63)	−0.53 (−0.66 to −0.40) .000	8.57 (8.48 to 8.66)
2023/25	−0.47 (−0.59 to −0.34) .000	8.49 (8.39 to 8.58)	−0.39 (−0.54 to −0.24) .000	8.61 (8.49 to 8.74)	−0.39 (−0.54 to −0.24) .000	8.61 (8.49 to 8.74)	−0.37 (−0.53 to −0.21) .000	8.62 (8.49 to 8.75)	−0.39 (−0.55 to −0.23) .000	8.62 (8.49 to 8.75)	−0.64 (−0.84 to −0.44) .000	8.52 (8.36 to 8.69)
*N*	6902		6239		6229		5909		5752		5046	

a Model 1 included age and sex as covariates; model 2 added educational attainment, last-known occupational position, and cognitive leisure activities to model 1; model 3 added marital status to model 2; model 4 added smoking, alcohol consumption, physical inactivity, and Mediterranean diet adherence to model 3; model 5 added cardiovascular biomarkers to model 4; and model 6 added fatigue, general self-rated health, grip strength, and chair stand test time to model 5.

There were no significant interaction effects when including an interaction term between time and age group or sex (eTable 4 in Supplemental Material).

## Discussion

In this Swiss population-based study, we observed a small but significant decline in cognitive performance over the past 20 years. These findings contrast with previous reports of improvements or stability in cognitive function [11–13], but concur with several other epidemiological studies [14–17], which observed a decline in cognitive performance and/or an increase in cognitive impairment. Modifiable dementia risk factors associated with poorer clock test performance included having a primary education level, a manual occupational position, low social contact, obesity, diabetes, and hypertension, while protective factors included high cognitive activity and high physical activity; but these factors did not explain the observed decline. Measures of physical function were also associated with cognitive performance but, while cognitive performance declined over time, there were improvements in some measures of physical function–notably chair rise time.

Diverging trends in cognitive performance across studies could be due to differences in sample characteristics, measures of cognitive function, study period, and/or study design. Our finding does not seem to be specific to the clock test as previous studies found a decline in performance on the MMSE, as well as other measures of perceptual speed, and verbal fluency [14–16]. Nevertheless, the trend may vary across different cognitive domains as one study found that the decline in MMSE performance was not observed for the Trail Making Test (B-A) of cognitive flexibility [14], while another study found the decline was stronger for perceptual speed than episodic memory [16]. It is not always possible to administer a full neuropsychological battery in cohort studies. Our study provides information on a populational level using a pragmatic screening tool for cognitive decline. Future research should aim to assess several cognitive domains in more detail to gain further insight into cognitive performance trends. Reports of improvements in cognitive function over time are more likely to occur in longitudinal than repeated cross-sectional studies if the former do not control for test experience [17].

To our knowledge, this is the first study examining associations between the LIBRA2 index and clock test performance. Consistent with previous research on other cognitive outcomes [6, 7], higher-risk LIBRA2 scores were associated with poorer clock test performance. The association between the LIBRA2 score and clock test performance was stronger than the associations for the individual factors (apart from education); although it would likely be even stronger had we included information on all 15 risk and protective factors. Modifiable dementia risk factors were associated with cognitive performance, but they did not explain the observed decline, suggesting that other factors play a role. It has been suggested that a decline in cognitive performance may be driven by a decreasing motivation to engage in effortful cognitive activities (referred to as need for cognition) [14, 30]. In our study, we did not see a secular change in the time spent engaging in cognitive activities over time. However, our measure did not capture all cognitive activities, and it will be important to examine the contribution of technology use to the observed trends, which may enhance or hinder cognitive function in the general population [31].

The association between physical and cognitive performance shown in previous studies was also apparent in the present study [29, 32]. However, there were some diverging trends in physical and cognitive performance. Consistent with another cohort study in Switzerland [14], there were improvements in timed physical performance (i.e. chair stand test), but no clear improvement in grip strength, which does not involve speed-related skills. Similarly, Santoni *et al.* [33] reported improvements in chair stand test performance and gait speed from 2001 to 2016, but no significant change in balance test performance. Improvements in speed-related physical performance alongside a decline in cognitive performance may reflect an adaptation of the population to modern digitalised societies. Recent studies in computational neuropsychiatry suggest that slowness of cognition combined with fast reactions may be an adaptive strategy in a fast-paced world [34].

In this study, the decline in cognitive performance cannot be explained by the COVID-19 pandemic as it was observed from 2011 to 2013 and stabilised after 2019. Data were not collected during the pandemic, but given that previous research reported cognitive decline from 2020 to 2022 [9], it is possible that performance declined further during this period before returning to 2019 levels. Similarly, there was no significant decline in mental health from pre- to post-pandemic in the subsample with depression data. This may be because mental health had returned to pre-pandemic levels following the pandemic, as indicated in previous research [35]. The increased digitalisation of society, notably the use of smartphones, could be an explanation for the trends observed in this study. Smartphones largely took over from feature phones in 2011–13, and the use of social media has increased exponentially. A recent meta-analysis showed a consistent, strong positive association between natural uses of digital technologies and overall cognitive well-being [31]. However, while moderate screen time might be cognitively stimulating, excessive passive screen time can be cognitively harmful [36]. Social media use in particular may increase memory failures in adults of any age [37], and may impair impulse control [38]. As excessive social media and smartphone use is generally more prevalent in younger age groups, it is possible that cognitive performance declines are even greater during young adulthood and adolescence, which is a critical period for cognitive development. On the other hand, the combination of older age with excessive media use may accelerate normal age-related cognitive decline. Future research should examine the contribution of smartphone and social media use to declines in cognitive performance across age groups.

Further research is needed to examine the contribution of mental health to the observed trends in cognitive performance. Among our subsample with depression data, there was no significant association between depression and clock test performance. This is perhaps surprising as depression is a well-established risk factor for dementia and cognitive impairment [5]. However, several studies show that depressed patients do not differ from controls in terms of their clock drawing performance—especially if the depression is early-onset versus late-onset [39]. It is possible that other mental health factors, such as stress, distractibility, or anxiety, partly explain the declining trends in clock test performance, and this should be explored in future research.

### Strengths and limitations

Study strengths include analysis of cognitive and physical performance measures alongside multiple modifiable risk factors for dementia across a 20-year period. To our knowledge, this is the first Swiss population-based study to examine both cognitive performance trends and the contribution of modifiable dementia risk factors. The data were from adults aged 50 years and above, but further insights could be gained from the inclusion of younger subgroups. Moreover, while we used an objective, reliable, and valid measure of cognitive performance, use of multiple cognitive measures and/or the qualitative clock test scoring system could provide insight into trends across cognitive domains. While we assessed multiple modifiable dementia risk factors, we did not have information on some risk factors, including sleep, hearing/vision loss, and recent illness. We used marital status as a proxy of social contact as comprehensive measures of social connectedness have not yet been administered in the Bus Santé cohort. Marriage is associated with an increase in social networks, particularly in later life [40], and is a significant predictor of dementia risk, overall health, and longevity [22]. However, measures of social isolation, loneliness, and social networks are more optimal measures of social connectedness and should be incorporated in future research using the LIBRA index. Moreover, the role of certain modifiable factors for which the evidence is mixed, such as dietary patterns, needs to be further investigated. It is unlikely that the observed improvements in speed-related physical performance, but no clear improvement in grip strength, alongside a decline in cognitive performance are attributable to survival bias as several pre-pandemic studies observed similar trends among other representative samples of older adults [14, 33]. However, it is important to acknowledge that the study samples may not be representative in terms of all characteristics relevant to performance test outcomes. Longitudinal studies that control for practice effects are needed to provide further insight into cognitive performance trends and the role of modifiable dementia risk factors.

## Conclusions

In this population-based study of Swiss adults, we found a small but significant decline in cognitive performance over the last two decades. Well-known modifiable dementia risk factors were associated with cognitive performance, but did not fully explain this decline, suggesting other factors play a role. There is a need for ongoing monitoring of cognitive performance in the general population, alongside potential contributing factors.

## Supplementary Material

ckag046_Supplementary_Data

## Data Availability

Data are available upon reasonable request made to the corresponding author. Key points Cognitive performance declined between 2005 and 2025. Modifiable dementia risk factors were associated with poorer cognitive performance. The decline remained significant after adjustment for the risk factors. Ongoing monitoring of cognitive health and contributing factors in the general population is needed.
