# Supplementary material for: Twenty-year trends in cognitive performance and modifiable dementia risk factors: a Swiss population-based study
Source: Eur J Public Health. 2026 Mar 31;36(2):ckag046. doi: 10.1093/eurpub/ckag046 (PMC13038253; doi:10.1093/eurpub/ckag046)
Supplement: ckag046_Supplementary_Data [file ckag046_supplementary_data.docx]

**Supplemental material**

**Reliability of clock test scores**

**eTable 1.** Operationalisation of factors included in the LIBRA2 index in Bus Sante

**eTable 2.** Characteristics of Bus Sante participants with clock test scores during 2005 to 2025

**eTable 3.** Adjusted proportions of cognitive impairment over time for all models, with OR and 95% confidence intervals.

**eTable 4.** Effects of age group and sex on the association between time and clock test performance, with beta and 95% confidence intervals.

**eFigure 1.** Mean clock test scores and 95% confidence intervals from 2005 to 2025 stratified by modifiable dementia risk factors.

**eFigure 2.** Mean clock test scores and 95% confidence intervals from 2005 to 2025 stratified by health indicators.

**Reliability of clock test scores**

Intra-rater reliability was assessed using the intra-class correlation coefficient (ICC), Bland–Altman plots, and Cohen's kappa statistic on data from 50 randomly-selected tests (coded twice). The test scores were strongly correlated (ICC (95% confidence interval (CI))=0.96 (0.93-0.98)). Bland–Altman plots indicated that the mean difference between test scores was small in the total sample (0.08 points), and most values fell within the 95% limits of agreement. The classification of cognitive impairment (yes, no) from the two test scores showed that 94% were placed in the equivalent category with very good agreement (Cohen's kappa (95% CI)=0.87 (0.73-1.00)).

**eTable 1.** Operationalisation of factors included in the LIBRA2 index in Bus Sante

| **Factor** | **Operationalisation Bus Sante** | **Weight LIBRA2** |
| --- | --- | --- |
| High alcohol consumption | Self-reported intake >21 standard units per week | +1.0 |
| Coronary heart disease | Self-reported diagnosis of angina, atherosclerosis, or MI | +2.6 |
| High physical activity | ≥150 minutes of moderate phsyical activity a week | -1.9 |
| Chronic kidney disease | Data not available | +1.8 |
| Diabetes | Fasting plasma glucose ≥7.0 mmol/L or self-reported diagnosis of diabetes | +2.2 |
| Cholesterol | Total blood cholesterol ≥ 5 mmol/L and low density lipoprotein ≥ 3 mmol/L or self-reported diagnosis of hypercholesterolemia | +2.6 |
| Smoking | Self-reported smoking | +2.5 |
| Midlife obesity | BMI ≥30 | +2.2 |
| Midlife hypertension | Mean blood pressure ≥ 140/90 mmHg or self-reported diagnosis of hypertension | +1.1 |
| Healthy diet | Mediterranean diet score (excluding alcohol consumption), divided into tertiles (highest tertile) | -1.2 |
| Depression | Data only available for a subsample | +4.1 |
| High cognitive activity | Self-reported hours spent weekly on intellectual activities per week, divided in tertiles (highest tertile) | -3.0 |
| Hearing impairment | Data not available | +2.4 |
| Low social contact | Never married / lived as a couple | +2.1 |
| Sleep disturbances | Data only available for a subsample | +1.1 |
| Theoretical range |  | -6.1 to +25.8 |
| Range Bus Sante |  | -6.1 to +13.2 |

**eTable 2.** Characteristics of Bus Sante participants with clock test scores during 2005 to 2025.

|  | Total | 2005/10 | 2011/13 | 2014/16 | 2017/19 | 2023/25 | p-value |
| --- | --- | --- | --- | --- | --- | --- | --- |
|  | N=6,902 | N=1,701 | N=1,330 | N=1,226 | N=1,522 | N=1,123 |  |
| High cognitive activity |  |  |  |  |  |  | 0.009 |
| No | 67.4 (4,393) | 70.2 (1,176) | 65.0 (856) | 65.2 (788) | 67.0 (991) | 69.2 (582) |  |
| Yes | 32.6 (2,128) | 29.8 (499) | 35.0 (461) | 34.8 (420) | 33.0 (489) | 30.8 (259) |  |
| Low social contact |  |  |  |  |  |  | 0.17 |
| No | 91.7 (6,271) | 91.7 (1,556) | 92.8 (1,234) | 91.6 (1,123) | 91.9 (1,391) | 90.0 (967) |  |
| Yes | 8.3 (570) | 8.3 (141) | 7.2 (96) | 8.4 (103) | 8.1 (122) | 10.0 (108) |  |
| Smoking |  |  |  |  |  |  | 0.56 |
| No | 82.9 (5,663) | 82.8 (1,403) | 81.6 (1,083) | 83.3 (1,019) | 83.3 (1,259) | 84.0 (899) |  |
| Yes | 17.1 (1,165) | 17.2 (292) | 18.4 (245) | 16.7 (205) | 16.7 (252) | 16.0 (171) |  |
| High alcohol consumption |  |  |  |  |  |  | <0.001 |
| No | 87.9 (5,868) | 83.4 (1,377) | 85.6 (1,121) | 89.0 (1,070) | 89.6 (1,328) | 94.3 (972) |  |
| Yes | 12.1 (808) | 16.6 (275) | 14.4 (188) | 11.0 (132) | 10.4 (154) | 5.7 (59) |  |
| Healthy diet |  |  |  |  |  |  | <0.001 |
| No | 68.8 (4,595) | 65.1 (1,075) | 67.9 (889) | 67.3 (809) | 67.8 (1,005) | 79.2 (817) |  |
| Yes | 31.2 (2,081) | 34.9 (577) | 32.1 (420) | 32.7 (393) | 32.2 (477) | 20.8 (214) |  |
| High physical activity |  |  |  |  |  |  | <0.001 |
| No | 33.4 (2,308) | 37.8 (643) | 35.1 (467) | 33.3 (408) | 32.6 (496) | 26.2 (294) |  |
| Yes | 66.6 (4,594) | 62.2 (1,058) | 64.9 (863) | 66.7 (818) | 67.4 (1,026) | 73.8 (829) |  |
| Obesity |  |  |  |  |  |  | 0.88 |
| No | 83.4 (5,753) | 83.2 (1,415) | 82.9 (1,103) | 83.1 (1,019) | 84.3 (1,282) | 83.2 (934) |  |
| Yes | 16.6 (1,146) | 16.8 (285) | 17.1 (227) | 16.9 (207) | 15.7 (239) | 16.8 (188) |  |
| Diabetes |  |  |  |  |  |  | 0.96 |
| No | 88.6 (6,112) | 88.5 (1,504) | 89.2 (1,186) | 88.5 (1,085) | 88.3 (1,343) | 88.5 (994) |  |
| Yes | 11.4 (787) | 11.5 (195) | 10.8 (144) | 11.5 (141) | 11.7 (178) | 11.5 (129) |  |
| Hypertension |  |  |  |  |  |  | <0.001 |
| No | 54.7 (3,772) | 50.0 (850) | 53.1 (706) | 56.0 (686) | 57.5 (875) | 58.5 (655) |  |
| Yes | 45.3 (3,127) | 50.0 (851) | 46.9 (624) | 44.0 (540) | 42.5 (647) | 41.5 (465) |  |
| High cholesterol |  |  |  |  |  |  | <0.001 |
| No | 21.6 (1,444) | 17.9 (287) | 16.6 (218) | 21.1 (257) | 28.7 (423) | 23.8 (259) |  |
| Yes | 78.4 (5,251) | 82.1 (1,315) | 83.4 (1,097) | 78.9 (960) | 71.3 (1,051) | 76.2 (828) |  |
| Coronary heart disease |  |  |  |  |  |  | 0.50 |
| No | 92.8 (6,337) | 93.0 (1,576) | 91.8 (1,221) | 93.6 (1,147) | 92.8 (1,404) | 92.7 (989) |  |
| Yes | 7.2 (493) | 7.0 (119) | 8.2 (109) | 6.4 (78) | 7.2 (109) | 7.3 (78) |  |
| Fatigue |  |  |  |  |  |  | 0.11 |
| Not at all | 47.4 (3,036) | 49.4 (838) | 47.5 (632) | 46.9 (574) | 44.7 (676) | 49.3 (316) |  |
| A bit | 44.6 (2,853) | 43.8 (742) | 45.0 (598) | 45.0 (551) | 45.4 (687) | 42.9 (275) |  |
| A lot | 8.0 (514) | 6.8 (116) | 7.5 (100) | 8.1 (99) | 9.8 (149) | 7.8 (50) |  |
| Self-rated health |  |  |  |  |  |  | 0.35 |
| Very good | 24.0 (1,643) | 24.6 (417) | 22.3 (296) | 25.9 (317) | 24.0 (363) | 22.8 (250) |  |
| Good | 55.1 (3,779) | 54.6 (926) | 56.2 (748) | 52.4 (641) | 55.0 (832) | 57.7 (632) |  |
| Average | 18.3 (1,255) | 17.8 (302) | 19.4 (258) | 18.7 (229) | 18.4 (278) | 17.2 (188) |  |
| Bad or very  bad | 2.6 (181) | 3.0 (51) | 2.1 (28) | 3.0 (37) | 2.6 (40) | 2.3 (25) |  |
| Grip strength, pounds | 70.1 (24.8) | 71.5 (24.5) | 68.8 (25.6) | 69.0 (24.4) | 70.1 (24.2) | 70.8 (25.5) | 0.016 |
| Chair rise time, seconds | 10.0 (3.4) | 11.3 (3.6) | 10.6 (3.4) | 10.1 (3.5) | 9.6 (2.9) | 8.0 (2.8) | <0.001 |

**eTable 3.** Adjusted proportions of cognitive impairment^1^ over time for all models, with OR and 95% confidence intervals.

|  | **Model 1** | | **Model 2** | | **Model 3** | | **Model 4** | | **Model 5** | | **Model 6** | |
| --- | --- | --- | --- | --- | --- | --- | --- | --- | --- | --- | --- | --- |
| **Time** | OR [95% CI]  p | Proportion [95% CI] | OR [95% CI] | Proportion [95% CI] | OR [95% CI] | Proportion [95% CI] | **Time** | OR [95% CI]  p | Proportion [95% CI] | OR [95% CI] | Proportion [95% CI] | OR [95% CI] |
| **2005/10 (REF)** |  | 0.14  [0.13,0.16] |  | 0.14  [0.12,0.16] |  | 0.14  [0.12,0.16] |  | 0.14  [0.12,0.16] |  | 0.14  [0.12,0.16] |  | 0.13  [0.11,0.15] |
| **2011/13** | 1.55  [1.28,1.87]  0.000 | 0.20  [0.18,0.23] | 1.57  [1.29,1.90]  0.000 | 0.20  [0.18,0.23] | 1.58  [1.30,1.92]  0.000 | 0.20  [0.18,0.23] | 1.55  [1.27,1.89]  0.000 | 0.20  [0.18,0.22] | 1.56  [1.27,1.91]  0.000 | 0.20  [0.18,0.22] | 1.77  [1.42,2.22]  0.000 | 0.20  [0.18,0.22] |
| **2014/16** | 1.63  [1.35,1.98]  0.000 | 0.21  [0.19,0.24] | 1.65  [1.36,2.02]  0.000 | 0.21  [0.19,0.23] | 1.66  [1.36,2.02]  0.000 | 0.21  [0.19,0.23] | 1.67  [1.37,2.05]  0.000 | 0.21  [0.19,0.24] | 1.69  [1.38,2.08]  0.000 | 0.21  [0.19,0.24] | 1.95  [1.55,2.44]  0.000 | 0.22  [0.19,0.24] |
| **2017/19** | 2.12  [1.78,2.53]  0.000 | 0.26  [0.24,0.28] | 2.18  [1.82,2.62]  0.000 | 0.26  [0.24,0.28] | 2.19  [1.82,2.63]  0.000 | 0.26  [0.24,0.28] | 2.13  [1.76,2.58]  0.000 | 0.25  [0.23,0.28] | 2.17  [1.78,2.63]  0.000 | 0.25  [0.23,0.28] | 2.42  [1.94,3.02]  0.000 | 0.25  [0.23,0.27] |
| **2023/25** | 2.28  [1.89,2.76]  0.000 | 0.27  [0.25,0.30] | 2.17  [1.73,2.71]  0.000 | 0.26  [0.22,0.29] | 2.18  [1.74,2.73]  0.000 | 0.26  [0.22,0.29] | 2.11  [1.66,2.68]  0.000 | 0.25  [0.22,0.29] | 2.16  [1.69,2.75]  0.000 | 0.25  [0.22,0.29] | 3.04  [2.24,4.13]  0.000 | 0.28  [0.23,0.33] |
| **N** | 6902 | | 6239 | | 6229 | | 5909 | | 5752 | | 5046 | |

^1^Clock test score of ≤7

Model 1 included age and sex as covariates; model 2 added educational attainment, last-known occupational position, and cognitive leisure activities to model 1; model 3 added marital status to model 2; model 4 added smoking, alcohol consumption, physical inactivity, and Mediterranean diet adherence to model 3; model 5 added cardiovascular biomarkers to model 4; and model 6 added fatigue, general self-rated health, grip strength, and chair stand test time to model 5.

**eTable 4.** Effects of age group and sex on the association between time and clock test performance, with beta and 95% confidence intervals.

|  | **b** | **95% CI** | **p-value** |
| --- | --- | --- | --- |
| Age group |  |  |  |
| 50-54 (REF) |  |  |  |
| 55-59 | -0.24 | [-0.48,-0.01] | 0.043 |
| 60-64 | -0.18 | [-0.41,0.06] | 0.136 |
| 65-69 | -0.13 | [-0.38,0.12] | 0.306 |
| 70+ | -0.34 | [-0.61,-0.07] | 0.014 |
| 2005/10 (REF) |  |  |  |
| 2011/13 | -0.36 | [-0.63,-0.08] | 0.011 |
| 2014/16 | -0.35 | [-0.63,-0.08] | 0.013 |
| 2017/19 | -0.59 | [-0.86,-0.33] | 0.000 |
| 2023/25 | -0.37 | [-0.65,-0.08] | 0.011 |
| 55-59 x 2005/10 (REF) |  |  |  |
| 55-59 2011/13 | 0.36 | [-0.00,0.72] | 0.053 |
| 55-59 x 2014/16 | 0.22 | [-0.13,0.58] | 0.220 |
| 55-59 x 2017/19 | 0.29 | [-0.04,0.63] | 0.088 |
| 55-59 x 2023/25 | 0.04 | [-0.32,0.41] | 0.812 |
| 60-64 x 2005/10 (REF) |  |  |  |
| 60-64 2011/13 | 0.15 | [-0.22,0.51] | 0.430 |
| 60-64 x 2014/16 | 0.07 | [-0.31,0.44] | 0.721 |
| 60-64 x 2017/19 | 0.08 | [-0.26,0.43] | 0.646 |
| 60-64 x 2023/25 | -0.12 | [-0.49,0.26] | 0.538 |
| 65-69 x 2005/10 (REF) |  |  |  |
| 65-69 2011/13 | 0.07 | [-0.30,0.44] | 0.693 |
| 65-69 x 2014/16 | 0.16 | [-0.22,0.54] | 0.414 |
| 65-69 x 2017/19 | 0.13 | [-0.24,0.49] | 0.494 |
| 65-69 x 2023/25 | -0.20 | [-0.60,0.21] | 0.336 |
| 70+ x 2005/10 (REF) |  |  |  |
| 70+ x 2011/13 | 0.39 | [-0.01,0.78] | 0.054 |
| 70+ x 2014/16 | 0.33 | [-0.08,0.75] | 0.110 |
| 70+ x 2017/19 | 0.16 | [-0.22,0.54] | 0.409 |
| 70+ x 2023/25 | 0.12 | [-0.30,0.54] | 0.576 |
| Male (REF) |  |  |  |
| Female | -0.06 | [-0.21,0.10] | 0.496 |
| Female x 2005/10 (REF) |  |  |  |
| Female x 2011/13 | 0.05 | [-0.19,0.29] | 0.662 |
| Female x 2014/16 | -0.10 | [-0.34,0.15] | 0.446 |
| Female x 2017/19 | 0.05 | [-0.18,0.28] | 0.691 |
| Female x 2023/25 | -0.14 | [-0.39,0.11] | 0.277 |
| N | 6902 |  |  |


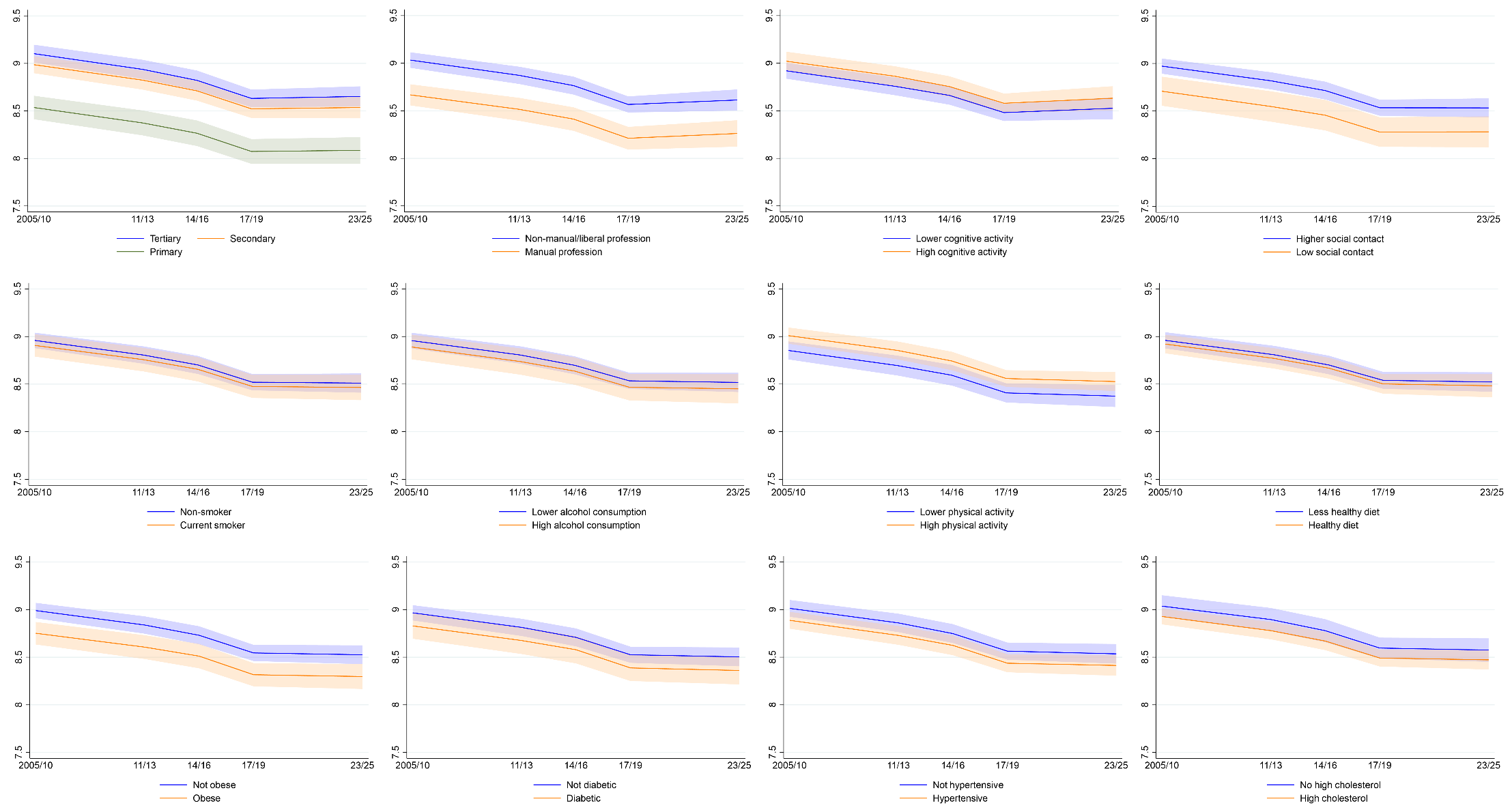


**eFigure 1.** Mean clock test scores and 95% confidence intervals from 2005 to 2025 stratified by modifiable dementia risk factors.

Models adjusted for age and sex.


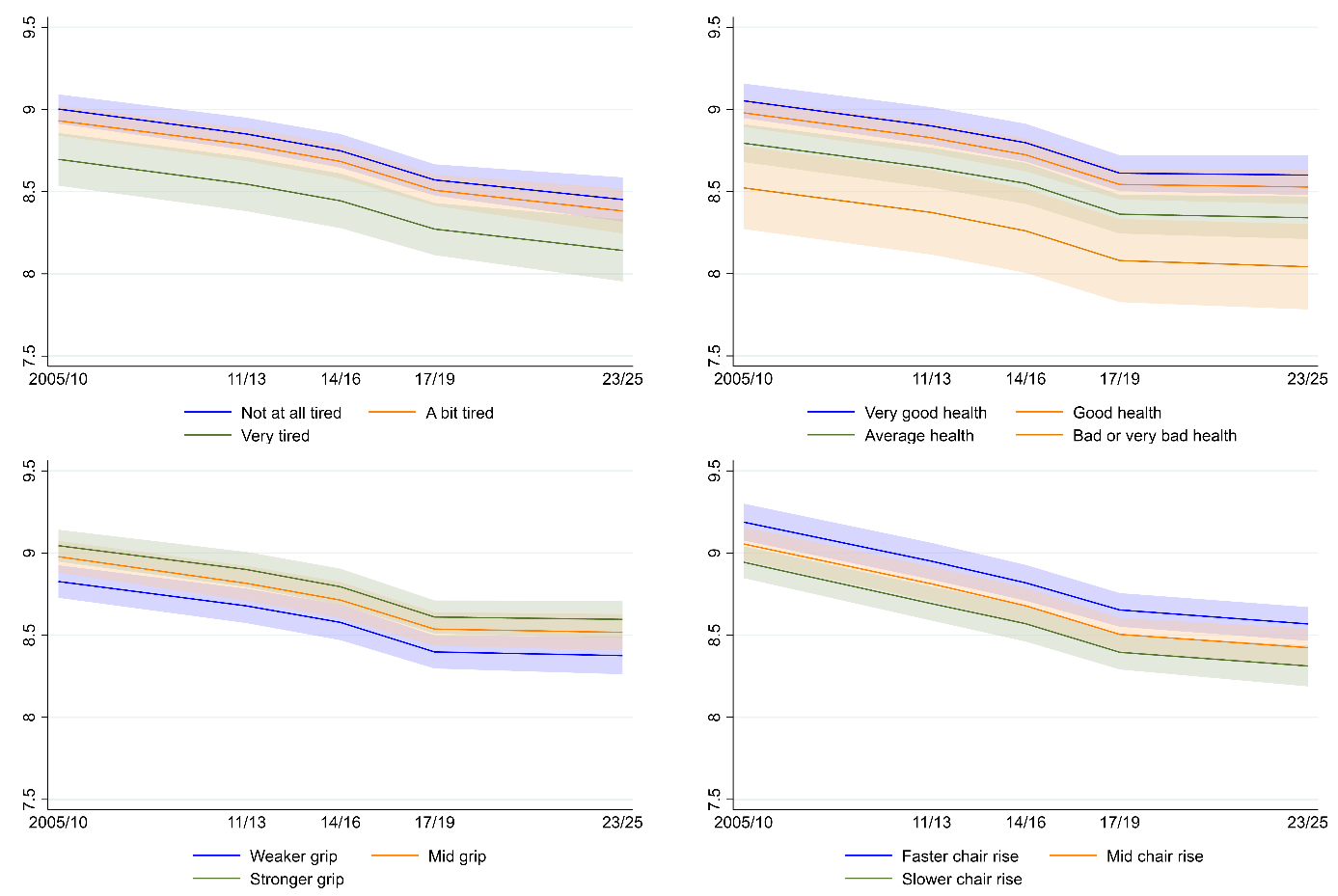


**eFigure 2.** Mean clock test scores and 95% confidence intervals from 2005 to 2025 stratified by health indicators.

Models adjusted for age and sex.
